# Variously Prepared Zeolite Y as a Modifier of ANFO

**DOI:** 10.3390/ma15175855

**Published:** 2022-08-25

**Authors:** Łukasz Kuterasiński, Agnieszka M. Wojtkiewicz, Marta Sadowska, Paulina Żeliszewska, Bogna D. Napruszewska, Małgorzata Zimowska, Mateusz Pytlik, Andrzej Biessikirski

**Affiliations:** 1 Jerzy Haber Institute of Catalysis and Surface Chemistry, Polish Academy of Sciences, 8 Niezapominajek Street, 30-239 Krakow, Poland; 2 Conformity Assessment Body, Central Mining Institute, 1 Gwarków Square, 40-166 Katowice, Poland; 3 Faculty of Civil Engineering and Resource Management, AGH University of Science and Technology, 30 Mickiewicz Avenue, 30-059 Krakow, Poland

**Keywords:** zeolite Y, ANFO, modifier, detonation, post-blast fumes

## Abstract

In the presented research, we investigated Ammonium Nitrate Fuel Oil (ANFO), with the addition of variously modified zeolite Y as an attractive explosive. Analysis of both blasting tests and thermodynamic models of blasting properties led to the conclusion that the addition of zeolite Y enhanced the detonation properties of such prepared ANFO via the growth of the detonation pressure, temperature, compression energy, and heat of the explosion. Generally, the modification of ANFO with variously prepared zeolite Y also reduced the volume of (CO_x_ + NO_x_) post-blast fumes. Furthermore, it was found that the ANFO’s velocity of detonation (VOD) could be controlled by the choice of the way of zeolite Y modification. Namely, for zeolite Y without Mg, as well as Mg-Y prepared via the impregnation method, the VOD rose. The opposite effect was observed when ANFO was modified with Mg-Y, obtained from the deposition of Mg over zeolite Y via the ultrasonic-assisted procedure.

## 1. Introduction

Explosives are currently widely used both in the military industry and in civil works, including mining, demolition works, or macro-leveling [1,2]. One of the most commonly used explosives (especially in mining) is ANFO (Ammonium Nitrate Fuel Oil) due to its easy and cheap procedure of production, as well as its good blasting properties [3,4].

ANFO is manufactured by the blending of ammonium nitrate(V) (AN) (oxygen-bearing component) with fuel oil (FO) (combustible component) in an appropriate mass ratio (usually 94:6). The ratio of 94:6 (AN:FO) provides a zero-oxygen balance, i.e., no excess or deficiency of oxygen in the balance of the composition of the explosive, which is responsible for the formation of maximal detonation energy with simultaneous low toxicity of the post-blast fumes (a minimal content of NO_x_ and CO), and reduced production of CO_2_ being responsible for greenhouse effect [5]. ANFO is considered a non-ideal explosive due to its non-ideal detonation behavior, which can be defined by the impossibility of the reach of its theoretically calculated values, such as the velocity of detonation (VOD) [6].

In mixed-type explosives (like ANFO), the oxygen-bearing component should be characterized by high porosity and low density. That allows to obtain a high value of the adsorption coefficient between the solid phase (AN) and the liquid phase (FO). The presence of a combustible component is necessary for the occurrence of explosive transformation [5,6,7,8,9,10,11,12,13,14].

Oxley et al. [15,16,17] reported a dual mechanism of the AN decomposition, i.e., according to a radical and an ionic pathway. The former type of mechanism is characterized by both a high rate and temperatures, whereas the latter route of reaction takes place at a low temperature with a low rate. In turn, the application of modifiers allows preparing explosives with optimized blasting properties, such as the velocity of detonation or explosion heat and strength. Furthermore, the addition of certain modifiers to ANFO may catalyze the decomposition of explosives, reduce the emission of post-blast fumes, and influence explosive sensitivity. However, the addition of a new component (acting as either a flammable ingredient or modifier) may cause a change in the oxygen balance [15,16,17].

Apart from FO [18,19,20,21,22,23,24,25,26,27,28,29,30,31,32,33,34,35,36,37,38,39,40,41,42,43,44], in the available bibliography, many examples of ANFO additives have been reported, such as: charcoal; coal dust; activated carbon [18,19,20,21,22,23,24,25,26]; 2,4,6-trinitrotoluene (TNT) [19]; nitromethane [27]; iron persulfide (II) [19]; pyrite [28,29]; aluminum, magnesium, copper and zinc dusts [5,20,21,30,31]; sugar [5,20,21]; lubricants [32]; polyolefin wastes [33,34,35,36]; polyethylene (PE) [37]; polypropylene (PP) [37]; sodium sulfate [38]; sodium decarbonate [16]; potassium carbonate [16]; ammonium sulfate [16]; calcium oxide [20,21]; dolomite [20,21]; anhydrite [20,21]; aluminum oxides [30,39]; and silica [40,41].

So far, in the present works dedicated to ANFO, only results concerning physicochemical and blasting properties of the systems consisting of AN mixed with oil, as well as with other organic and inorganic modifiers mainly in the form of dust and inorganic salts, were reported. However, the use of additives acting as a carrier for inorganic modifiers has not been published. An example of this type of additive could be zeolite due to the presence of, among others, silicon, and aluminum in the zeolite framework, and due to the possibility of introducing a wide range of other elements to this group of minerals.

In the undertaken research, we investigate zeolite Y due to its low silicon-to-aluminum molar ratio (2 < Si/Al < 5), which means that the zeolite with this topology contains a lot of aluminum and therefore is characterized by a high ion exchange capacity, in comparison with the majority of zeolites of other structures. Relative high aluminum content (and automatically introduced metals to the zeolite) allows the use of this mineral as representative support for inorganic ANFO modifiers. The introduced metal into zeolite Y will be magnesium, due to the beneficial effect of this element on the blasting properties of ANFO, which has been published in one of our previous works [31].

We also try to find an answer to the question of whether the choice between (a) ion exchange, (b) impregnation, or (c) ultrasonic irradiation, as methods of the introduction of magnesium to zeolite skeleton, may affect the usefulness of zeolite Y as the carrier of inorganic modifiers in ANFO.

In the present paper, we reported the synthesis of ANFO consisting of AN, FO, and a modifier being the Y-structure type zeolite containing magnesium, which was incorporated to zeolite via three various routes (Mg-Y), or without magnesium. Separate studies are listed for the bare ANFO sample (without zeolite additive), which plays the role of the reference sample. The obtained explosives were subjected to physicochemical characterization including crystallinity/structure (XRD, FT-IR), surface/morphology (AFM/SEM), thermal properties (TG/DSC), and blasting properties (involving velocity of detonation, the heat of the explosion, compression energy, oxygen balance and the analysis of post-blast fumes).

## 2. Materials and Methods

### 2.1. Materials and Sample Preparation

Ammonium Nitrate porous prill (UltrAN 70) was produced in 2021 by Yara’s International A SA (Szczecin, Poland). UltrAN 70 contained ca. 34.5% nitrogen. The prill diameter and bulk density were in the range of 1.0–2.0 mm and 670–720 kg/m^3^, respectively. The moisture did not exceed 0.3% wt. The product was characterized by a purity of 99.4%.

Fuel oil (FO) was manufactured in 2021 by Silesia Oil Sp. z o.o. (Łaziska Górne, Poland) and consisted of C10-C20 hydrocarbons. The bulk density and kinetic viscosity of FO at 40 °C were 800 kg/m^3^ and 13.6 mm^2^/s, respectively. The detailed characterization of FO can be found in [42]. The reference sample ANFO (designated as “A”) was prepared by a blending of UltrAN 70 with FO according to the weight ratio of 94.0:6.0 (Table 1). The procedure was conducted by Yara’s International A SA.

The zeolite with Y-type structure (Si/Al = 2.65) was supplied by Mątwy (Inowrocław, Poland). The parent zeolite was in sodium form. The zeolite Y was added to the ANFO via a simple mixing and played the role of the enhancer of its blasting properties. Depending on the weight loading of the zeolite in the resulting ANFO-type material (1% wt. vs. 2% wt.), the sample was denoted as B1 and B2, respectively (Table 1).

The parent zeolite (Na-Y) was also modified with magnesium. The introduction of Mg into zeolite Y was performed via wet impregnation (Mg-Y_impr_), ion-exchange (Mg-_Yion-exch_), or ultrasonic-assisted impregnation procedure (Mg-Y_son_), respectively. A detailed description of the production of Mg-containing zeolites (Mg-Y) was given in Supplementary Materials.

ANFO-based materials, prepared via the addition of Mg-Y_impr_, Mg-Y_ion-exch,_ and Mg-Y_son_ zeolites to ANFO (via the blending route), were designated as C1 or C2, D1 or D2, and E1 or E2. Digits “1” and “2” refer to the percentage weight contribution of zeolite in the obtained ANFO. Details on the ANFO’s synthesis procedure were summarized in Table 1.

### 2.2. Characterization Methods

The crystallinity of the ANFO samples in the granule form was measured using the X-ray diffraction (XRD) phenomena with a PANalytical X’Pert PRO MPD diffractometer (40 kV, 30 mA), equipped with a CuKα generator (λ = 1.5418 Å). The experiments were conducted for a 2θ angle at 5–50° with a 0.033° step.

Structural analysis of the synthesized materials was performed using a Nicolet iS10 spectrometer (Thermo Scientific, Waltham, MA, USA) equipped with an MCT detector in the Attenuated Total Reflectance (ATR) mode. The FT-IR analysis was performed at 4000–650 cm^−1^, with a resolution of 4 cm^−1^ and the number of scans during a single measurement was 64.

The morphology of the obtained ANFO was investigated using a Jeol JSM-7500F scanning electron microscope (JEOL Ltd., Tokyo, Japan) equipped with the X-ray energy dispersive (EDS) system INCAPentaFETx3 (JEOL Ltd., Tokyo, Japan). Two detectors were used, and the images were recorded in two modes; the secondary electron detector provided SEI images and the backscattered electron detector provided BSE (COMPO) micrographs. Directly before SEM mapping, the samples were dried for 24 h and coated with chromium (20 nm).

The status of the surface of the studied samples was investigated using an atomic force microscope (AFM) NT-MDT Solver BIO apparatus (Moscow, Russia) equipped with the SMENA SFC050L scanning head. All measurements were conducted in air and in semicontact mode by the application of high-resolution silicon probes (NT-MDT ETALON probes, HA NC series, polysilicon cantilevers with resonance frequencies 140 kHz +/− 10% or, 240 kHz +/− 10% force constants 4.4 N/m +/− 20% or, 9.5 N/m +/− 20%, respectively, a typical curvature radius of the tip was 10 nm and cone angle was less than 20°). Obtained images were referred to the randomly chosen areas over the substrate and within the scan area suitable for the tested samples. All images were flattened and graphically developed using specialized software supplied with the instrument.

Thermal analysis of the prepared samples included Thermogravimetry (TG) and Differential Scanning Calorimetry (DSC). The measurements were performed using NETZSCH STA 409 PC/PG equipment (Netzsch-Gerätebau, Selb, Germany) at 20–700 °C. The temperature ramp was 10 °C/min. All studied samples were kept in airflow (30 mL/min) both in the furnace and the balance chamber. Air functioned as a stimulator of detonation conditions. For all measurements, the prepared sample (20 mg) was placed in the DSC aluminum pan using a spatula. To obtain the correct TG baseline, the same heating profiles were used for both the empty pan and the pan with a measured sample. The TG drift was ca. 5 μg, which corresponds to 0.02 mass%.

The velocity of detonation (VOD) was determined according to the procedure reported in [43]. Briefly, the ANFO charge (600 g) was placed in a glass tube of 46.4 mm inner diameter. The Royal Demolition Explosive (RDX-based) charge (14 g) initiated the detonation. Two short circuit probes were placed throughout the measured ANFO sample. The distance between probes was 150 mm. Moreover, the distance between the primer and the first probe was equal to twice the charge diameter (l). A crucial parameter in this measurement was the time difference (t) between the first recorded signal change in each probe. VOD can be defined according to Equation (1):

VOD = l/t(1)

Post-blast fumes analysis was conducted according to the procedure described in [44], which was based on regulations reported in [45]. The description of blasting tests has also been reported in our previous works [41,46,47]. In each experiment, 600 g of non-ideal ANFO charge was placed inside the steel mortar locked in the blasting chamber. After the sealing of the blasting chamber, the detonation of the explosive charge was performed. After that, the homogenization of the post-blast fumes took place. The homogenization duration was 3 min. Afterward, post-blast gases were collected in the ventilation system for 20 min. The CO_x_ (CO + CO_2_), as well as NO_x_ volumes, were measured using IR (MIR 25e) and chemiluminescent (TOPAZE 32M) analyzers, respectively. The measurement errors of VOD and post-blast fumes analyses were 2% and 1%, respectively.

Thermodynamic calculations were conducted in the EXPLO5 software produced by OZM Research with a calculation error of 5%. Calculations were made based on Becker-Kwiatkowski-Wilson (BKW) equation state [48]. EXPLO5 software included the calculations of parameters, among others, the explosion pressure and temperature, heat of the explosion, compression energy, and oxygen balance.

## 3. Results and Discussion

### 3.1. Structure

**Figure 1:**
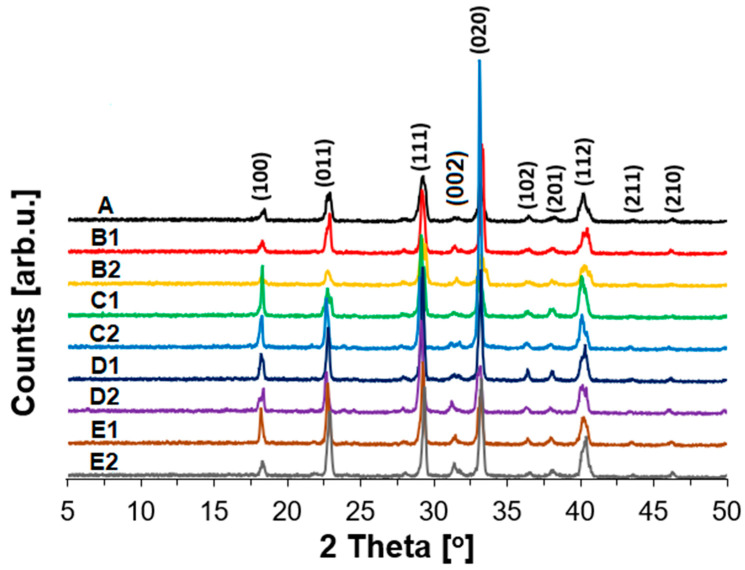
XRD patterns of the studied ANFO samples.

From the appearance of XRD images given in Figure 1 for all studied samples, the presence of Ammonium Nitrate in the orthorhombic crystal system and with a Pmmm space group and two molecules per unit cell was confirmed. Based on bibliography, the diffraction peaks at 2θ = 18°, 23°, 29°, 31°, 33°, 36°, 38°, 40°, 43°, and 46° can be assigned to (100), (011), (111), (002), (020), (102), (201), (112), (211), and (210) reflections in ANFO crystallites, respectively [49,50,51,52].

The addition of variously modified zeolite Y to ANFO altered the intensity of reflections coming from AN. Nevertheless, neither the appearance of new reflexes coming from the zeolite phase nor apparent shift was found. Similar effects we observed when we modified ANFO with microstructured charcoal (MC), which was reported in [18]. In turn, other effects were reported by Xu et al. [51], who added organic potassium salts to AN. They showed that the application of this type of additive influenced AN due to the formation of hydrogen bonds by polar groups, which led to the interaction between ammonium and nitrate ions. Based on the results reported by Xu et al. [51], we can expect that the mixing of additives (in this case zeolite Y both in the form of Na-Y and Mg-Y) with AN could result in structural changes in respect to the bare AN. On the other hand, when we compare XRD patterns of variously modified zeolite Y (Figure S1) [53], we can see that some reflexes overlap with the XRD pattern of AN, which can influence the intensity of the reflections assigned to AN. Interestingly, the XRD signals coming from the zeolite phase (Figure S1), and not overlapping with AN reflections, are absent in XRD images obtained for the samples consisting of ANFO mixed with zeolite. At first sight, it could be explained by a low content of zeolite in the prepared materials (not exceeding 2% wt.), but in our previous work [41], we reported that the addition of 1 or 2% wt. of silica resulted in the appearance of a weak signal attributed to this additive.

**Figure 2:**
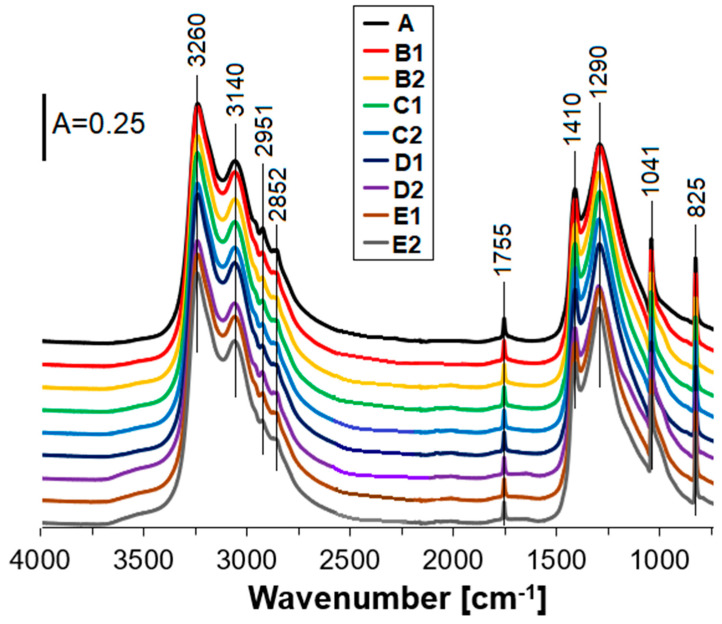
FT-IR spectra of the studied ANFO samples.

From FT-IR spectra recorded for all samples (Figure 2), the existence of the bands at 3260–2852 cm^−^^1^ is due to asymmetric vibrations of ammonium cation both in stretching and deformation modes. The maxima at 1755 cm^−^^1^ can originate from a stretching vibration or in-plane deformation of nitrate anion, or may result from a combination of an asymmetric deformation of NH_4_^+^ with a lattice mode. Apparent bands at 1410 and 1290 cm^−^^1^ can be attributed to the triply degenerated deformation of ammonium cation and the doubly degenerated NO_3_^−^ stretching vibration. In turn, the signals with the maxima at 1041 and 825 cm^−^^1^ reflect in-plane symmetric stretching and out-of-plane deformation of nitrate ion [49,50,54,55,56]. The bands at 2951 and 2852 cm^−^^1^ can be also due to -CH_2_- and -CH_3_ stretching vibrations from Fuel Oil [57]. Probably, the bands assigned to FO and AN skeletal vibrations overlapped each other.

The bands attributed to zeolite Y powder (referring to both 1% wt. and 2% wt. of the addition) were not detected. Direct comparison between the appearance of the FT-IR spectra for the prepared ANFO samples depicted in Figure 2, and the FT-IR spectra given in Figure S2 for variously modified zeolite Y samples, leads to the conclusion that the bands typical of AN structure overlapped with the signals coming from zeolite phase. Interestingly, the appearance of a small band at ca 1400 cm^−^^1^ for all Mg-Y samples can evidence the presence of magnesium in the form of MgO [58,59].

### 3.2. Morphology and Surface

**Figure 3:**
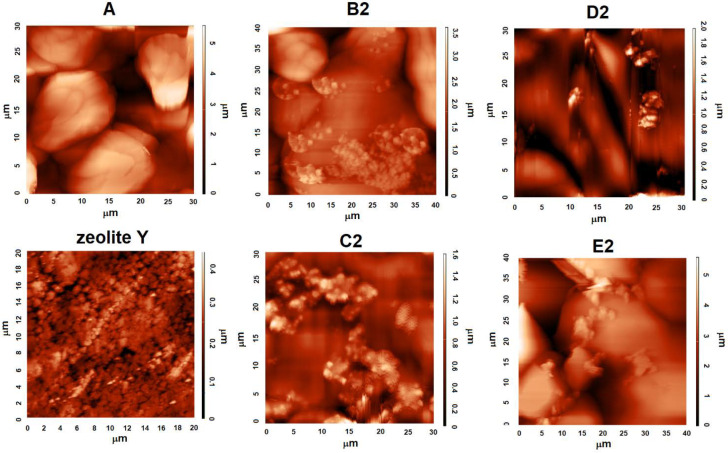
AFM images of the surface of ANFO samples.

**Figure 4:**
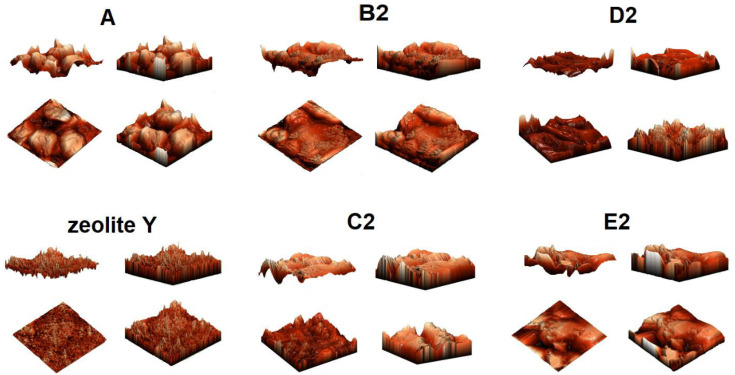
AFM visualization of the surface of ANFO samples in different projections.

AFM analysis (Figure 3 and Figure 4) indicated the presence of numerous surface deformations on AN prills in all studied samples. The presence of irregular-shaped AN grains are also apparent. The surface of the tested samples was hilly and was characterized by the existence of bulges. The height of the bulges was the highest for the ANFO without zeolite (for sample A) and reached 5 μm. For the ANFO samples containing 2% wt. of zeolite Y (either in Na-Y or Mg-Y forms)—samples B2, C2, D2, and E2—the occurrence of very small zeolite grains was detected. Another effect was the lowering of the bulge height to 2–3.5 μm.

Zeolite Y was characterized by another character of surface in comparison with ANFO-type materials (Figures S3 and S4). First of all, the surface of zeolite Y seemed to be much more uniform in relation to AN. Furthermore, the variously modified zeolite Y grains were small (with sizes of 1–3 μm and heigh below 1 μm) in respect to AN prills (with diameters in the range of 2–30 μm—Figure 3). The state of the zeolite Y surface depended clearly on the route of zeolite modification. The most uniform surface was found for Mg-Y prepared via the ion-exchange procedure, whereas the most differentiated surface was found for Mg-Y prepared using wet impregnation of zeolite Y with magnesium nitrate.

**Figure 5:**
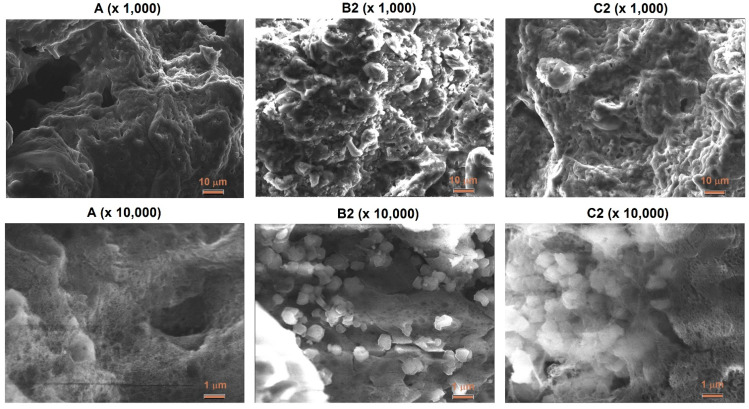
SEM images of ANFO-based samples.

SEM images of two magnifications (×1000 and ×10,000) illustrate the influence of the zeolite additive on the morphology of ANFO samples (Figure 5). Ammonium nitrate porous prill crystals mixed with Fuel Oil (sample A) are in the form of wrinkles, surface deformations, and cracks. The morphology of sample A resembles Chinese Cabbage, which is full of holes. The addition of zeolite Y (either with or without Mg) resulted in significant changes in the morphology of the prepared samples. First of all, the appearance of irregular-shaped crystals was found. This new crystalline phase undoubtedly comes from zeolite Y (Samples B2 and C2). Unfortunately, due to the very high sensitivity of the ANFO surface to the electron beam, we were not able to perform the SEM measurements for all prepared samples (D2 and E2). It was caused by the impossibility of achieving a vacuum (probably because of the decomposition of AN) and tearing the surface of the studied samples by the electron beam. Hence, we mainly used the AFM technique to characterize the surface of ANFO-type materials. However, the information obtained from SEM images is in line with the results taken from AFM analysis.

The influence of the way of the zeolite Y modification on its morphology was studied (Figure S5). It was indicated that independently of the zeolite Y modification (both in Na-Y and Mg-Y form), zeolite grains are of irregular shape with sizes smaller than 1 μm. No apparent differences in morphology between variously prepared zeolites were found.

### 3.3. Thermal Properties

**Figure 6:**
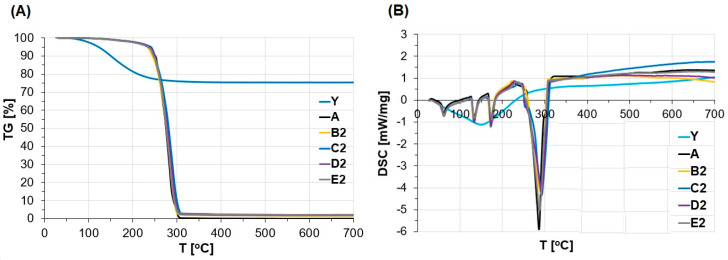
(**A**) TG and (**B**) DSC analysis of the ANFO samples.

To investigate the influence of the addition of variously modified zeolite Y on the thermal decomposition of ANFO, thermal analysis has been conducted. The TG mass loss and DSC heat effects were measured for ANFO containing 2% wt. of variously prepared zeolite Y (either without or with Mg introduced to zeolite via various techniques). The TG and DSC results are depicted in Figure 6A,B, respectively.

TG data (Figure 6A) showed that for all ANFO samples, a small mass loss (at a temperature lower than 250 °C) was caused by the evaporation of FO from crystalline Ammonium Nitrate. The evaporation of FO took place initially from the surface pores, later from the FO monolayer, and then continued from further FO layers [15].

A further increase of the temperature to the region at 250–300 °C led to the decomposition of AN according to Equation (2):

NH_4_NO_3_→N_2_O + 2H_2_O (2)

That resulted in a total mass decrease of all measured samples. The effect was the same independently of the modification of ANFO with zeolite Y (both in Na-Y form and Mg-Y zeolite prepared via various routes).

For comparison, pure zeolite Y demonstrated high thermal stability. Observed mass loss (22%) resulted from the desorption of water from the surface and then from internal parts of zeolite grains. A relatively high amount of removed water from zeolite can be explained by the high hydrophilicity of zeolite Y, which is typical of zeolites with a low Si/Al molar ratio [60].

DSC curves, obtained at 20–700 °C for all prepared ANFO-type materials, are illustrated in Figure 6B. In order to facilitate interpretation, separated TG/DSC data given for each studied sample were illustrated in Figures S6–S10. The DSC profiles indicate four endothermic peaks at 60, 135, 175, and 287–293 °C. Two signals at ca. 60 and ca. 135 °C originate from the crystallographic transformation of AN III→II and AN II→I, respectively [61]. A third endothermic signal at ca. 175 °C can be attributed to the AN melting point. Furthermore, rising temperature led to the thermal decomposition of AN, which was confirmed by the appearance of the fourth DSC peak with a maximum at ca. 287–293 °C [46,61]. From the analysis of DSC profiles, it may be concluded that the modification route of ANFO with zeolite Y has a small influence on the decomposition temperature of prepared non-ideal explosives. For example, ANFO without zeolite (sample A) undergoes decomposition at 287 °C with a −5.82 mW/mg thermal effect. The addition of zeolite Y containing 2% wt. of Mg introduced via the ultrasonic treatment of aqueous Mg precursor (sample E2) resulted in the decrease of DSC peak to −5.02 mW/mg without the change of peak maximum (at 287 °C). When ANFO was modified with 2% wt. of zeolite without Mg (B2), an energetic effect decreased to −4.25 mW/mg with the simultaneous growth of decomposition temperature to 293 °C. Similar observations took place in the case of the samples prepared via the blending of ANFO with 2% wt. of Mg-Y prepared via wet impregnation (C2) and ion-exchange (D2), for which thermal effects were −4.32 mW/mg and −4.20 mW/mg, respectively. In turn, DCS curve maxima were at 293 °C in both cases.

In our recent work [47], we reported that the maximum of DSC peak responsible for the decomposition of AN can depend on both the size of the microstructured charcoal (MC) grains and the chemical composition of the prepared samples. Generally, higher MC content and smaller MC grains lowered ANFO decomposition temperature (from 292 to 272 °C). In our case, we did not investigate the influence of zeolite additive grains, because we used zeolite in the form of powder.

In another work [62], we reported that the AN provenance (AN as fertilizer: AN-F vs. AN porous prill used in the mining industry: AN-PP) has a profound impact on the temperature of AN decomposition. AN-PP (applied in the undertaken research) is characterized by a lower decomposition temperature.

For comparison with another bibliography, not connected with our previous research, Fedroff et al. [63] reported that AN undergoes decomposition above 230 °C. Nevertheless, AN can deflagrate above 325 °C. Depending on the critical diameter, Ammonium Nitrate can explode at 260–300 °C.

### 3.4. Detonation Properties

The results of a quantitative analysis of post-blast fumes and velocity of detonation (VOD) measured for the prepared non-ideal explosive samples are summarized in Table 2. Total CO_x_ and NO_x_ post-blast volume was equal to 133.4 dm^3^ from 1 kg of tested charge (sample A). Generally, the addition of zeolite caused a slight decrease of (CO_x_ + NO_x_) volume to 126.0–131.6 dm^3^/kg depending on either the zeolite loading (% wt.) in the ANFO sample or the route of zeolite preparation. One exception was sample E2, for which cumulative (CO_x_ + NO_x_) volume was higher (133.8 dm^3^/kg) in respect to the sample without zeolite additive (A).

The most apparent effects were observed for the ANFO containing 1% wt. (B1) and 2% wt. of zeolite Y in sodium form (B2), for which cumulative CO_x_ and NO_x_ volumes were equal to 130.7 dm^3^/kg and 126.0 dm^3^/kg, respectively. In this case, we can see that a higher amount of zeolite additive in the ANFO sample caused a lowering in the formation of CO_x_ and NO_x_. In the case of ANFO modified with the zeolite Y containing Mg, we observed another effect. First of all, when we used the ANFO containing 2% wt. of Mg-Y zeolite (samples C2, D2, and E2), we detected higher total emission of CO_x_ and NO_x_ (129.8 dm^3^/kg, 131.6 dm^3^/kg and 133.8 dm^3^/kg, respectively) than for the analogs modified with 1% wt. of Mg-Y (sample C1—129.1 dm^3^/kg, sample D1—129.3 dm^3^/kg and sample E1—130.9 dm^3^/kg). At first sight, the presented results seem to be ambiguous. However, the analysis of EDS results (Table S1) allows us to notice that a rising weight Mg/Na ratio resulted in higher production of CO_x_ and NO_x_ from the detonation of 1 kg of explosive. Hence, it seems that the presence of Na in zeolite additive has a positive impact on the reduction of post-blast fumes during detonation of ANFO, whereas Mg evokes an opposite effect. From the analysis of the data summarized in Table S1, we can also establish that the weight ratio between Mg and Na in zeolite additive depends strictly on the way of the preparation of Mg-Y zeolite. The modification of ANFO with Mg-Y, in which Mg was introduced to zeolite via ion-exchange procedure (samples D1 and D2) or sonochemical-assisted technique (samples E1 and E2) resulted in a higher Mg/Na weight ratio followed by higher cumulative production of post-blast fumes.

When we focus only on the emission of CO_2_, we can see that for all zeolite-containing samples (B1, B2, C1, C2, D1, D2, E1, and E2), the addition of Na-Y or Mg-Y zeolite resulted in a slight reduction of CO_2_ among post-blast gases from 121.7 dm^3^/kg (sample A) to 114.1–119.5 dm^3^/kg. The lowest emission of CO_2_ was found for sample B2 (ANFO modified with 2% wt. of Na-Y zeolite).

Our results on the blasting properties of the studied explosives were compared with the research reported in the available bibliography. However, it was impossible to make a direct comparison between our research described in the current work and other ANFO + zeolite mixtures due to the lack of a bibliography dedicated to the application of zeolites in the manufacturing of ANFO.

Maranda et al. [21] reported that increasing amounts of Al powder caused a distinct reduction of the toxic fumes formed during detonation. In turn, in our recent work [41], we reported that the application of silica affected the oxygen balance of the non-ideal explosive towards positive values, which can be explained by the reduction of FO content and the additional introduction of oxygen (existing in the silica). The shift towards positive oxygen balance caused a high increase in the NO_x_ and CO_2_ volume. It is worth emphasizing that zeolites also belong to oxide materials, and thus should be a source of oxygen. Hence, zeolites can influence the oxygen balance, and we observed an increase in NO_x_ production among post-blast fumes. On the other hand, minimal reduction of CO_x_ can be explained by the presence of Al in the structure of the zeolite.

In another previous work [47], we reported the application of microstructure charcoal (MC) powders. In this case, the addition of MC to ANFO also caused the growth of the CO_X_ and NO_X_ volume among post-blast gases due to the additional oxygen which was present in the charcoal, and which was reported in the XPS analysis, but which was not included in the calculations. Similar to zeolites and silica, in this case, the shifting oxygen balance toward positive values also takes place. This shift is due to the occurrence of oxygen in the MC chemical composition, which resulted in the MC oxygen balance being lower than assumed at the beginning. That led to the changes in the chemical composition of the studied ANFO + MC systems [47]. Muzyk and Topolnicka [64] indicated that oxygen content decreased with the rank of coal. For example, in flame coal, coke, and anthracite, the oxygen content was at 2–17%. In brown coals, the oxygen amount is in the range of 15–35%.

Our findings concerning the increase of NO_x_ among post-blasting gases are in agreement with the results reported by Bhattacharyya et al. [65]. Besides the excess of oxygen in the explosive chemical composition or application of the additive which has a positive oxygen balance, the production of NO_x_ depends on the ANFO’s grain size, the amount of FO added to Ammonium Nitrate, as well as the mixing between AN and FO [65,66,67,68,69,70]. Bhattacharyya et al. [65] indicated that detonation of finer-grained ANFO caused a higher concentration of NO_x_ in post-blast oxides in relation to the coarser-grained analog, which may be explained by a faster reaction of finer-grained ANFO and a higher oxygen volume available to the chemical reaction leading to a more intensive formation of NO_x_. Opposite effects were observed by Sapko et al. [66], who reported that the use of powdered ANFO limited the emission of NO_x_ fourfold than usual ANFO. Observed phenomena can be explained by a higher mix between fuel and powdered AN prill, leading to a fuller AN decomposition and a higher VOD. On the other hand, it was shown that the application of ANFO of lower FO content caused the growth of NO_x_ in post-blast fumes, whereas an excess of FO added to AN resulted in less NO_x_ content among post-blast oxides due to a better stoichiometric blending between AN and FO [67,68,69,70].

Experimental data concerning the velocity of detonation (VOD) indicated a clear correlation between VOD values and the type of used ANFO. For ANFO without zeolite (sample A), VOD was 2024 m/s. In the case of ANFO modified with Na-Y zeolite, VOD grew from 2024 m/s to 2129 m/s (for sample B1) and to 2176 m/s (for sample B2). A notable increase in VOD was also observed for ANFO samples modified with Mg-Y prepared via the impregnation method, for which VOD increased to 2096 m/s (for sample C1) and 2122 m/s (for sample C2). An opposite effect was observed for the ANFO modified with Mg-Y via the ultrasonic-assisted procedure. For samples E1 and E2, VOD decreased from 2024 m/s to 1945 m/s and 1981 m/s, respectively. An ambiguous effect was observed for the ANFO modified with Mg-Y zeolite obtained via the ion-exchange procedure. In the case of the sample containing 1% wt. of zeolite (D1), VOD was reduced from 2024 m/s to 1947 m/s, meanwhile, explosive containing 2% wt. of zeolite demonstrated VOD equaling to 2078 m/s. In all cases, VOD increased with the zeolite loading in the tested explosive. Based on the presented results of the blasting properties of the prepared ANFO, it can be concluded that the VOD values of the studied explosives can be easily controlled by both the zeolite Y content and the choice of the zeolite Y modification way. The density of the prepared ANFO samples depended weakly on their route of preparation. For the reference sample (A), density was 695 kg/m^3^, while for the ANFO containing zeolite (B1, B2, C1, C2, D1, D2, E1, and E2), density was slightly lower and was in the range of 671–686 kg/m^3^.

The results on the VOD values are comparable with our previous studies [71], however, VOD should rise linearly with ANFO’s density. In turn, in our undertaken study, we have a similar density of the prepared samples, thus the VOD values are also in a relatively narrow range (1947–2176 m/s). The separate subject for a debate constitutes the influence of the modifier content on the VOD values. As mentioned above, generally, the VOD values increased with the zeolite content in ANFO. When we compared this tendency with the results reported for our previous research [41], where a growing amount of silica in ANFO caused ambiguous changes in VOD values, we stated the opposite effect. This discrepancy can be explained by some fundamental differences between silica and zeolite-type additive. Namely, silica is a typical inert component in ANFO, meanwhile, zeolite Y in the form of both Na-Y and Mg-Y can play a multifunctional role. Zeolite Y can be a source of oxygen (influencing oxygen balance), as well as it supports silicon, sodium, magnesium, and aluminum, being fuel for ANFO. This explanation can be confirmed by Miyake et al. [6], who investigated the velocity of detonation of explosives made of Ammonium Nitrate (AN) blended with activated carbon (AC). They indicated that in AN:AC systems VOD raised with AC content (AN:AC containing 5% wt. of AC was characterized by the VOD value reaching 3400 m/s). Similar observations took place in the case of the mixture of ANFO with microstructured charcoal (ANFO + MC), where the addition of MC to ANFO caused the increase of VOD from 1586 m/s to 1617–2046 m/s depending on both the amount of MC in (ANFO + MC) sample and the size of charcoal enhancer grain [47].

Calculated blasting properties are summarized in Table 3. Performed calculations indicated the potential impact of zeolite additive on the tested ANFO’s properties. All investigated parameters (detonation pressure, detonation temperature, heat of explosion, and compression energy defined in [48] being effects of detonation) rose with the loading of ANFO with variously modified zeolite Y. Detonation pressure grew from 3838 MPa for reference AMFO (sample A) to 3844–3966 MPa and to 3954–4126 MPa for samples containing 1% wt. or 2% wt. of zeolite, respectively. For both zeolite contents (1 and 2% wt.), more apparent growths in detonation pressure were indicated for the samples containing Mg-Y zeolite prepared via the ion-exchange or ultrasonic-assisted procedure. The values exceeding 4000 MPa were obtained only for ANFO blended with 2% wt. of zeolite containing magnesium introduced into zeolite Y via ion-exchange process (4008 MPa—sample D2) and using ultrasounds (4126 MPa—sample E2). When we compare our calculated detonation pressure values with EDS results (Table S1), we can see that a rising weight Mg/Na ratio favors a higher detonation pressure. Similar tendencies were found for the heat of the explosion and compression energy. For the heat of the explosion, we obtained rising values from 3913 kJ/kg (sample A) to 4352–4384 kJ/kg (samples B1–E1) and to 4785–4816 kJ/kg (samples B2–E2). In turn, the simulated values of compression energy grew from 795 kJ/kg (sample A) to 819–856 kJ/kg (samples B1–E1) and to 873–889 kJ/kg (samples B2–E2).

In the case of calculated detonation temperature, the addition of zeolite Y (both in the form of Na-Y and Mg-Y) to ANFO resulted in the increase of values from 2970 K (for sample A) to 3204–3224 K and to 3428–3443 K (for ANFO modified with 1% wt. and 2% wt. of zeolite Y, respectively), and was independent of the way of zeolite modification. Only one simulated parameter, which demonstrated such minor changes, was oxygen balance. The addition of variously modified zeolite Y (both in the form of Na-Y and Mg-Y) to ANFO caused very small shifts towards more positive values from −0.99% (for pure ANFO) to −0.98% and −0.97% (when ANFO contained 1% wt. or 2% wt. of zeolite, respectively).

The obtained calculations correspond to our previous research [31], where we reported that the addition of Al or Mg powder to ANFO resulted in significant growth of heat of explosion from 3940 kJ/kg (for pure ANFO) to 4510 J/kg (for ANFO + Al) and to 4500 kJ/kg (for ANFO + Mg), that can be explained by the role of Al and Mg as a fuel being an additional resource of energy [5,20,21,30,31,39]. Another cause of the elevated values of the detonation pressure, temperature, heat of the explosion, and compression energy, can be the afterburning effect, which is a phenomenon resulting from the secondary reaction between unreacted FO (and/or partially oxidized post-blast fumes coming from the primary reactions) with the surrounding air [72,73]. On the other hand, higher values of those parameters are in line with oxygen balance, which also increased and was close to zero.

In a total other view, when we treat zeolite as a system consisting of Si-O and Al-O groups (i.e., semimetal subsystems), we could assume this type of additive introduced to ANFO (being non-ideal composition) combusting behind the reaction front zone. That could lead to the increase in detonation temperature and pressure due to the growth of the surface being responsible for the heat exchange between the zeolite grains and the specific product formed during the reaction of the ANFO decomposition [39].

## 4. Conclusions

- In the present work, we presented the assessment of the blasting properties of Ammonium Nitrate Fuel Oil (ANFO) with the addition of variously modified zeolite Y.
- The presence of zeolite Y in ANFO did not change the structure, but altered ANFO’s surface, morphology, and influenced slightly thermal properties of such synthesized ANFO.
- The addition of zeolite Y to ANFO led to the growth of the detonation pressure, temperature, and heat of the explosion.
- We can control the VOD of ANFO by the choice of the way of the modification of zeolite Y additive. For bare zeolite Y and Mg-Y prepared via the impregnation method, the velocity of detonation (VOD) rose. The opposite effect was observed for ANFO modified with Mg-Y obtained from the deposition of Mg over zeolite Y via the ultrasonic-assisted procedure.
- The utilization of variously modified zeolite Y as an ANFO modifier generally reduced the volume of (CO_x_ + NO_x_) post-blast fumes, which is desired from an ecological point of view.

## Figures and Tables

**Table 1:** Chemical composition of non-ideal explosives and their synthesis conditions. The total sample mass was normalized to 5.00 g.

Sample	Chemical Composition [% wt.]			Description
	Ammonium Nitrate	Fuel Oil	Zeolite Y	
A	94.00	6.00	0.00	Commercial ANFO, reference sample (5.00 g)
B1	93.06	5.94	1.00	ANFO (4.95 g) + zeolite Y (0.05 g)
B2	92.12	5.88	2.00	ANFO (4.90 g) + zeolite Y (0.10 g)
C1	93.06	5.94	1.00	ANFO (4.95 g) + Mg-Y (0.05 g). Zeolite Y containing Mg introduced via the impregnation method.
C2	92.12	5.88	2.00	ANFO (4.90 g) + Mg-Y (0.10 g). Zeolite Y containing Mg introduced via the impregnation method.
D1	93.06	5.94	1.00	ANFO (4.95 g) + Mg-Y (0.05 g). Zeolite Y containing Mg introduced via the ion-exchange method.
D2	92.12	5.88	2.00	ANFO (4.90 g) + Mg-Y (0.10 g). Zeolite Y containing Mg introduced via the ion-exchange method.
E1	93.06	5.94	1.00	ANFO (4.95 g) + Mg-Y (0.05 g). Zeolite Y containing Mg introduced via ultrasonic-assisted impregnation method.
E2	92.12	5.88	2.00	ANFO (4.90 g) + Mg-Y (0.10 g). Zeolite Y containing Mg introduced via ultrasonic-assisted impregnation method.

**Table 2:** Post-blast fumes, the velocity of detonation (VOD), and density of non-ideal ANFO with the addition of zeolite Y.

Sample	CO_2_ [dm^3^/kg]	CO [dm^3^/kg]	NO_x_ [dm^3^/kg]	CO_x_ and NO_x_ Post-Blast Volume [dm^3^/kg]	VOD [m/s]	Density [kg/m^3^]
A	121.7	4.01	7.64	133.4	2024	695
B1	119.5	4.41	6.83	130.7	2129	683
B2	114.1	4.69	7.24	126.0	2176	672
C1	117.0	4.17	7.96	129.1	2096	676
C2	116.0	4.85	9.00	129.8	2122	671
D1	116.4	4.18	8.73	129.3	1947	684
D2	117.9	3.80	9.94	131.6	2078	676
E1	118.4	3.81	8.73	130.9	1945	686
E2	119.5	4.48	9.83	133.8	1981	686

**Table 3:** Calculated properties of non-ideal ANFO with the addition of variously modified zeolite Y.

Sample	Detonation Pressure [MPa]	Detonation Temperature [K]	Heat of Explosion [kJ/kg]	Compression Energy [kJ/kg]	Oxygen Balance [%]
A	3838	2970	3913	795	−0.99
B1	3871	3204	4352	819	−0.98
B2	3954	3428	4785	873	−0.97
C1	3844	3220	4381	835	−0.98
C2	3961	3443	4811	877	−0.97
D1	3966	3224	4379	856	−0.98
D2	4008	3441	4813	878	−0.97
E1	3900	3211	4384	821	−0.98
E2	4126	3441	4816	889	−0.97

## Data Availability

Not applicable.

## Supplementary Materials

The following supporting information can be downloaded at: https://www.mdpi.com/article/10.3390/ma15175855/s1, Figure S1: XRD patterns of the variously modified Y-type zeolites; Figure S2: FT-IR spectra of the variously modified Y-type zeolites; Figure S3: AFM images of the surface of variously modified Y-type zeolites; Figure S4: AFM visualization of the surface of variously modified Y-type zeolites in different projections; Figure S5: SEM images of variously modified Y-type zeolites; Figure S6: TG/DSC profiles for sample A; Figure S7: TG/DSC profiles for sample B2; Figure S8: TG/DSC profiles for sample C2; Figure S9: TG/DSC profiles for sample D2; Figure S10: TG/DSC profiles for sample E2; Table S1: EDS (Energy Dispersive Spectroscopy) chemical analysis of variously modified zeolite Y.
