# Supplementary material for: Variously Prepared Zeolite Y as a Modifier of ANFO
Source: Materials (Basel). 2022 Aug 25;15(17):5855. doi: 10.3390/ma15175855 (PMC9457274; doi:10.3390/ma15175855)
Supplement: Supplementary file 1 [file materials-15-05855-s001.zip › materials-1837986-supplementary.pdf]

materials

Supplementary Materials

Variously Prepared Zeolite Y as a Modifier of ANFO

Łukasz Kuterasiński [1,*], Agnieszka M. Wojtkiewicz [1], Marta Sadowska [1], Paulina Żeliszewska [1], Bogna D. Napruszewska [1], Małgorzata Zimowska [1], Mateusz Pytlik [2,*] and Andrzej Biessikirski [3,*]

[1] Jerzy Haber Institute of Catalysis and Surface Chemistry, Polish Academy of Sciences, 8 Niezapominajek Street, 30-239 Kraków, Poland
[2] Conformity Assessment Body, Central Mining Institute, 1 Gwarków Square, 40-166 Katowice, Poland
[3] AGH University of Science and Technology, Faculty of Civil Engineering and Resource Management, 30 Mickiewicz Avenue, 30-059 Kraków, Poland
* Correspondence: lukasz.kuterasinski@ikifp.edu.pl (Ł.K.); mpytlik@gig.eu (M.P.); abiess@agh.edu.pl (A.B.).

The modification of zeolite Y with Mg

a) impregnation (Mg-Y$_{impr}$)

6 g of a dry zeolite Na-Y (Inowrocław-Mątwy) was impregnated with a 5.33 g of $Mg(NO_3)_2$*$6H_2O$ (Sigma-Aldrich, ACS>99%). Afterward, the resulting slurry was dried overnight at 80 °C.

b) ion-exchange (Mg-Y$_{ion-exch}$)

A Fivefold Na^+/NH_4^+ ion-exchange procedure of zeolite Y (30 g) was conducted using 500 ml of 0.5 M aqueous magnesium nitrate at 80 °C for 2 h.

c) ultrasonic-assisted impregnation (Mg-Y$_{son}$)

In the ultrasonic method, zeolite Y (5g) was immersed in 0.5 M aqueous magnesium nitrate solution (200 ml) and treated with ultrasounds for 30 min using QSonica Q-700 sonicator (Church Hill Rd, Newtown, CT, USA) equipped with a "½" diameter horn (the average power of sonication was 60 W and frequency was 20 kHz). During the sonication procedure, the glass tube filled with the zeolite precursor was placed in an ice bath in order to maintain room temperature.

Final treatment: Mg-Y zeolites obtained according to procedures denoted as a), b) and c) subjected to a triple centrifugation (4200 RPM for 10 min), drying overnight at 80 °C and the calcination at 500 °C for 4 h in airflow (50 ml/min).

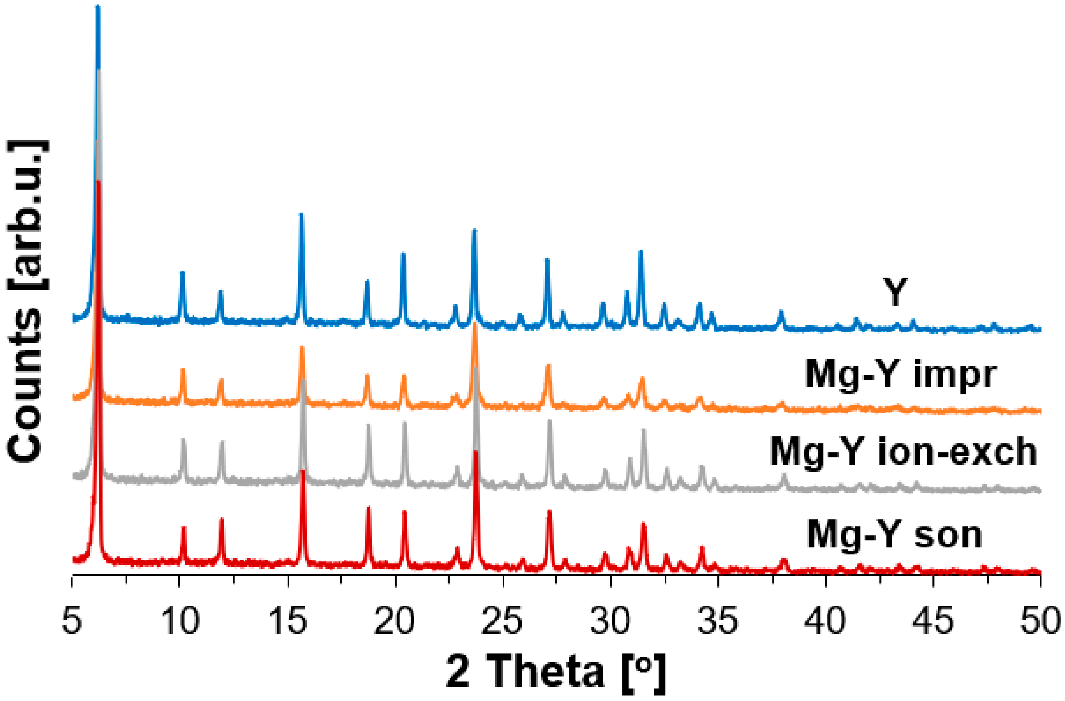

Figure S1. XRD patterns of the variously modified Y-type zeolites.

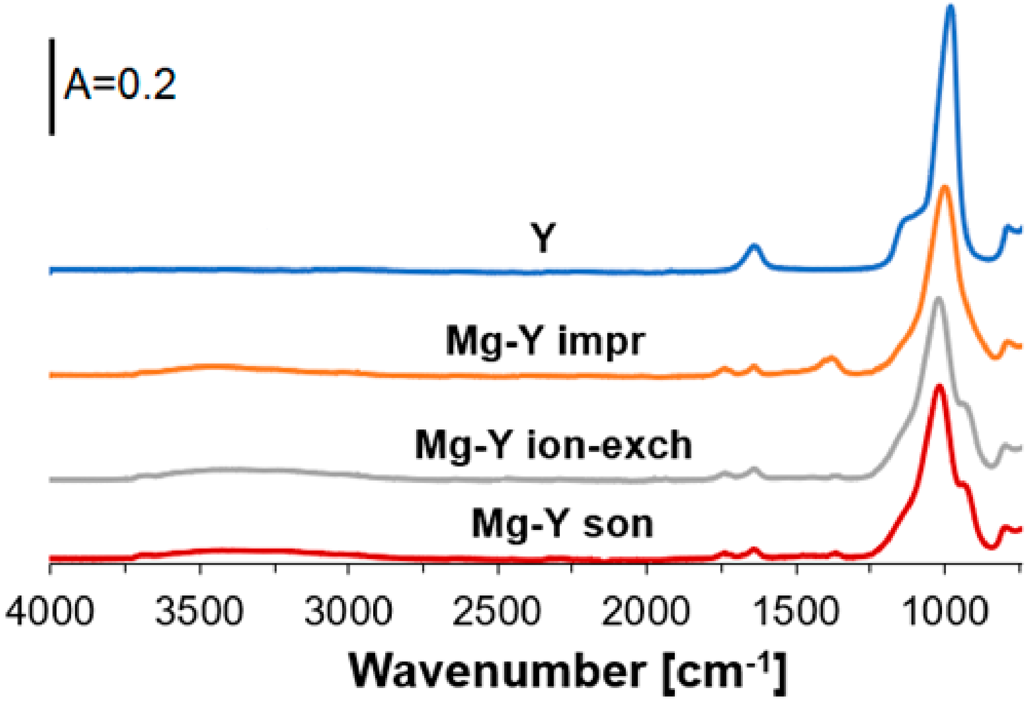

Figure S2. FT-IR spectra of the variously modified Y-type zeolites.

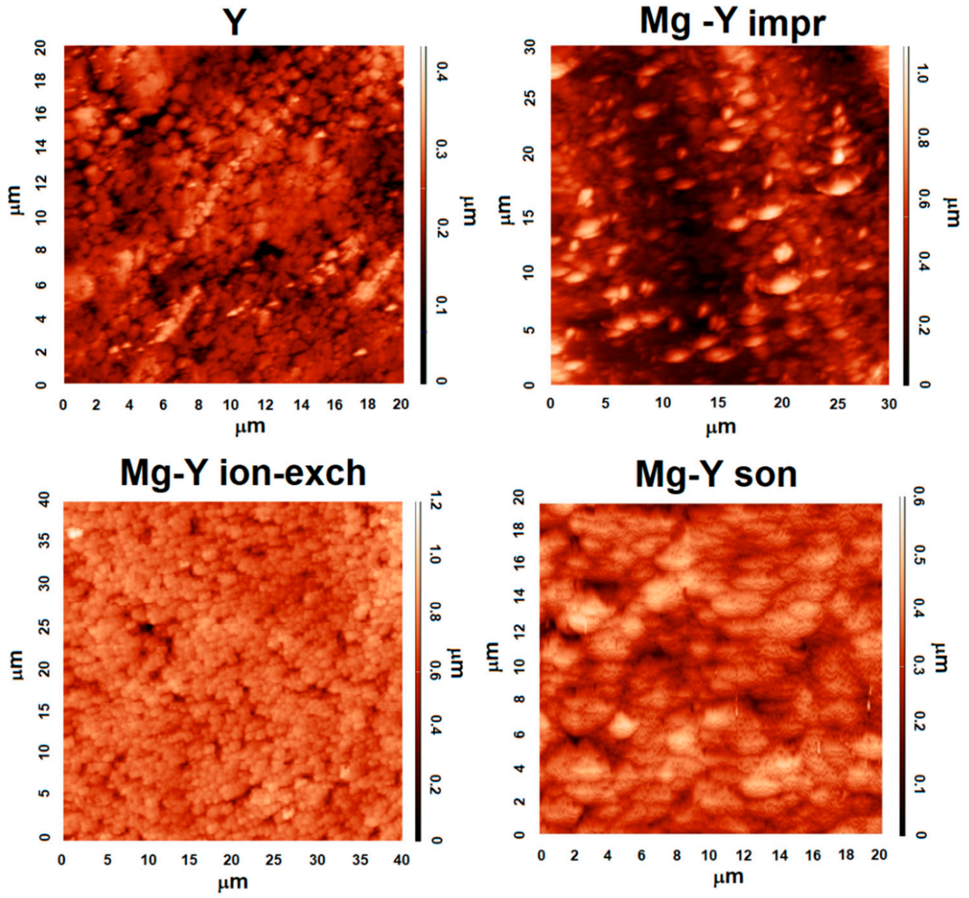

Figure S3. AFM images of the surface of variously modified Y-type zeolites.

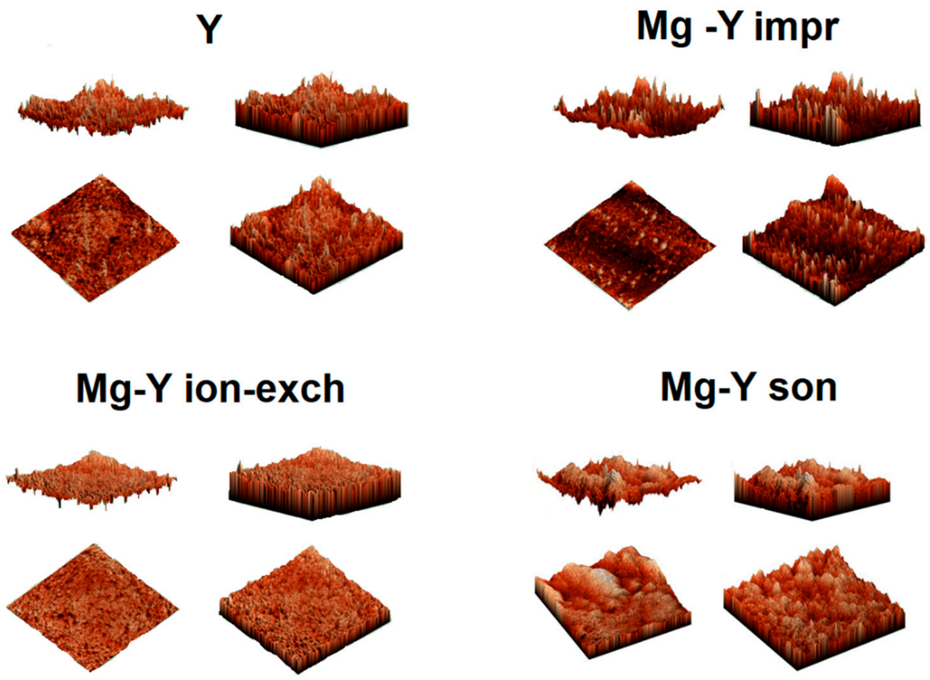

Figure S4. AFM visualization of the surface of variously modified Y-type zeolites in different projections.

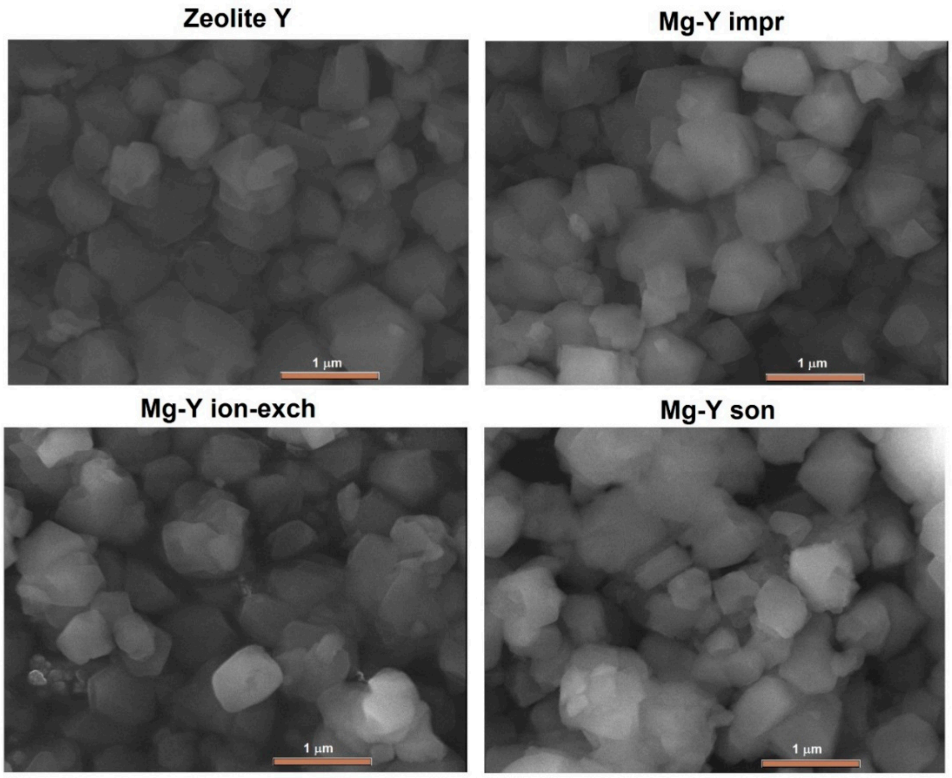

Figure S5. SEM images of variously modified Y-type zeolites.

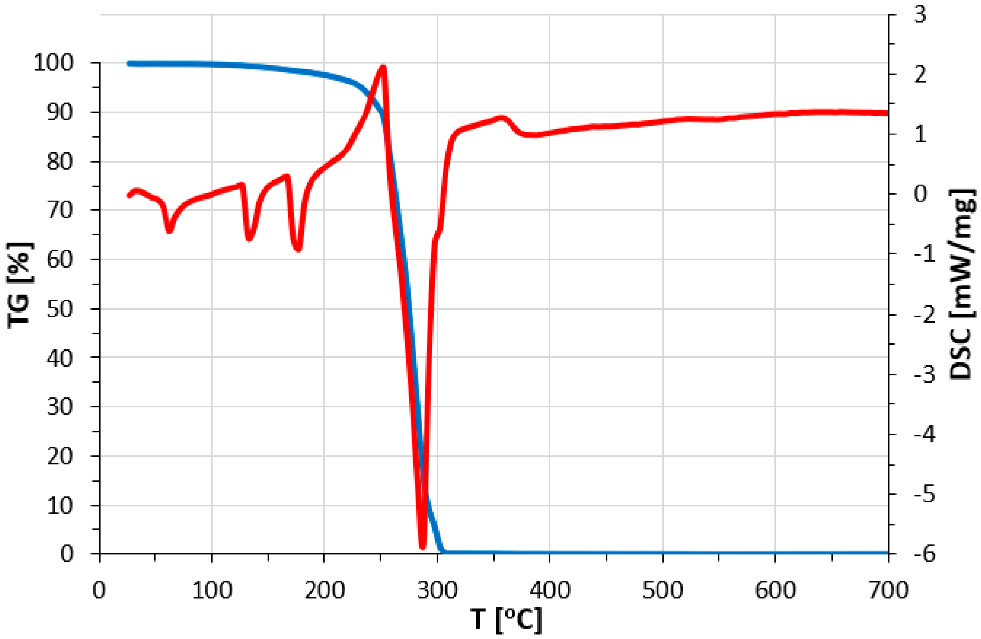

Figure S6. TG/DSC profiles for sample A.

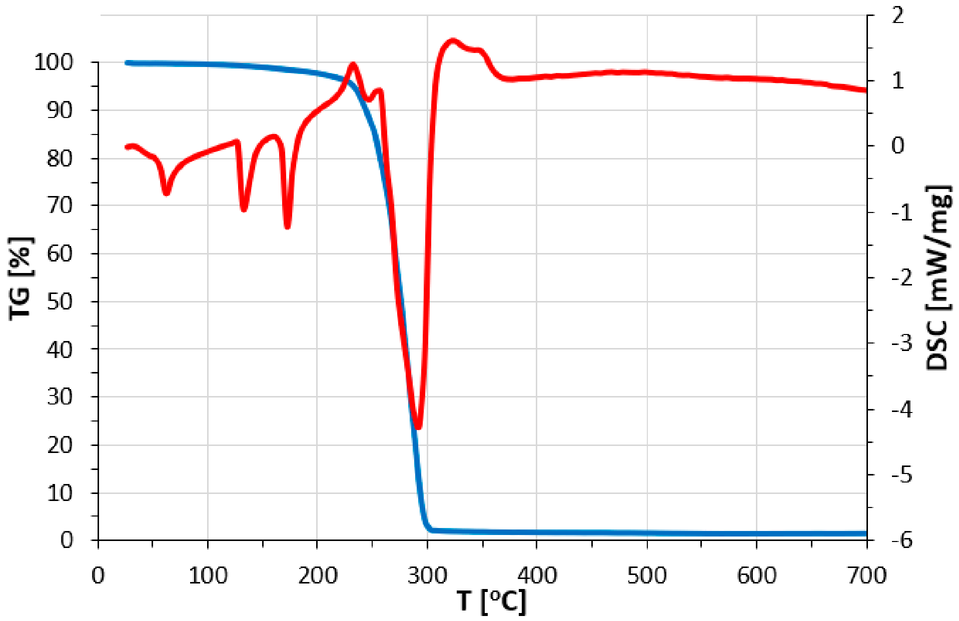

Figure S7. TG/DSC profiles for sample B2.

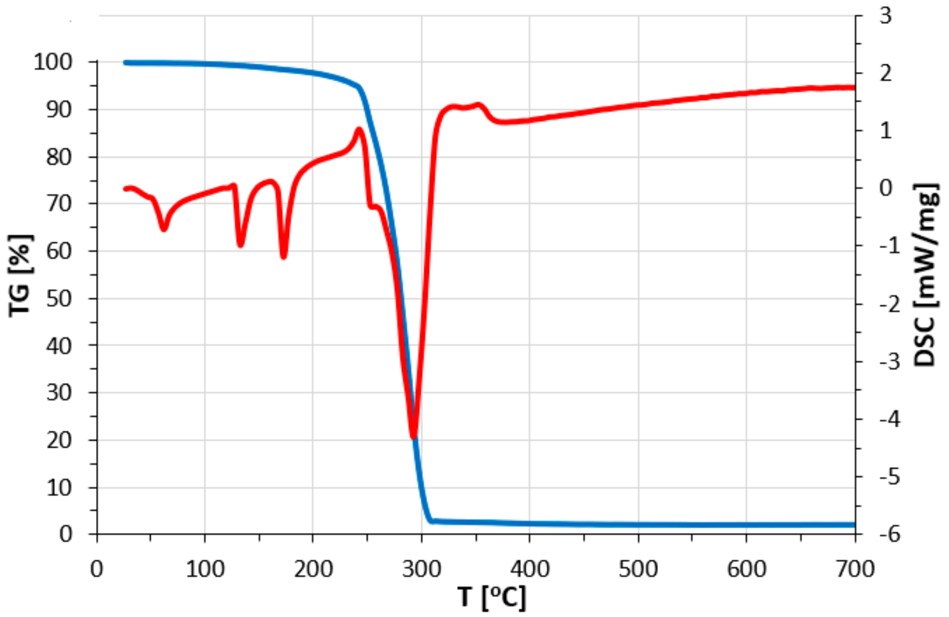

Figure S8. TG/DSC profiles for sample C2.

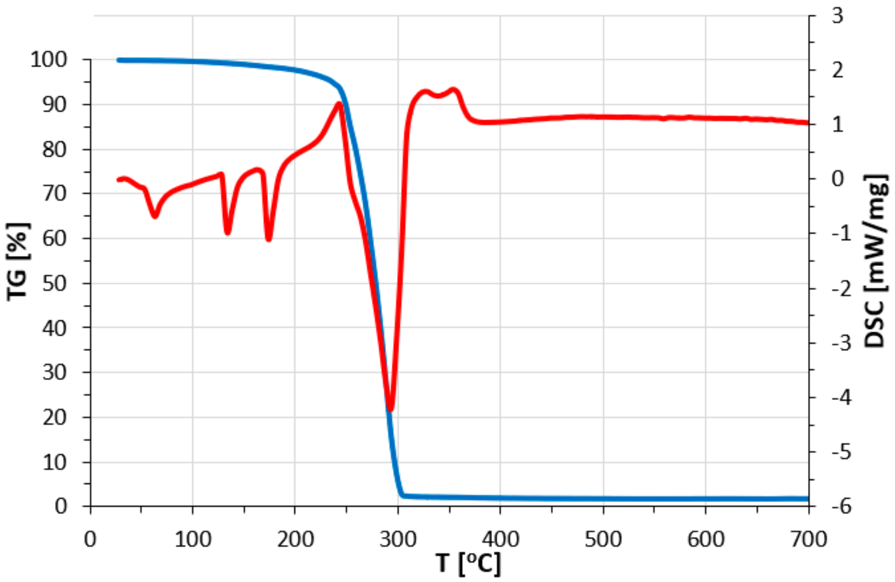

Figure S9. TG/DSC profiles for sample D2.

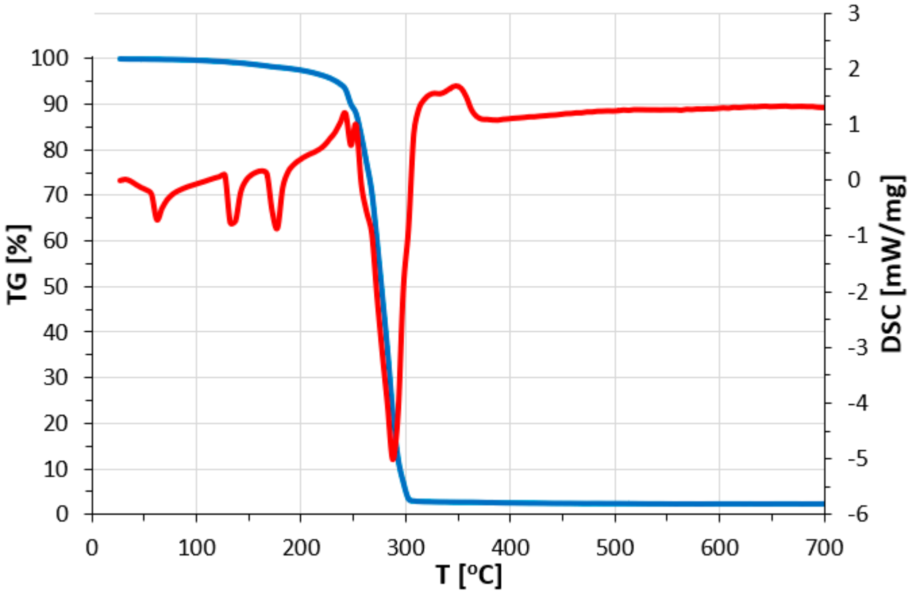

Figure S10. TG/DSC profiles for sample E2.

Table S1. EDS (Energy Dispersive Spectroscopy) chemical analysis of variously modified zeolite Y.

Sample	Chemical analysis (EDS)					
	Si [% wt.]	Al [% wt.]	O [% wt.]	Na [% wt.]	Mg [% wt.]	Mg [% wt.]/ Na [% wt.]
**zeolite Y**	21.5	7.9	65.6	5.0	0.0	0.0
**Mg-Y impr**	16.5	5.9	68.2	5.2	4.2	0.81
**Mg-Y ion-exch**	18.3	8.2	69.0	1.8	2.7	1.50
**Mg-Y son**	19.4	8.3	65.8	3.4	3.1	0.91
